# Comparative Analysis of Motor Preparation Using Bereitschaftspotential With Two Methods of Wrist Extension

**DOI:** 10.7759/cureus.79458

**Published:** 2025-02-22

**Authors:** Ashlesh Patil, Kanwal P Kochhar

**Affiliations:** 1 Physiology, All India Institute of Medical Sciences, Nagpur, Nagpur, IND; 2 Physiology, All India Institute of Medical Sciences, New Delhi, New Delhi, IND

**Keywords:** bereitschaftspotential, motor control, neurorehabilitation, precision of movement, supplementary motor area

## Abstract

Introduction: Motor control for voluntary movement involves the orchestrated activity of motor cortical areas, which activate approximately one to two seconds before movement onset. This preparatory neural activity, termed Bereitschaftspotential (BP), reflects the readiness of cortical circuits for initiating movement. BP has been linked to parameters such as force and rate of contraction; however, the influence of movement precision on BP components is less understood. This study aims to investigate how movement precision affects BP characteristics, providing insights into the neural dynamics underlying motor preparation.

Methods: Using a quasi-experimental design, BP characteristics were compared in 15 healthy, right-handed male participants (ages 40-60) under two conditions: precise wrist extensions (Method 1) and self-paced wrist extensions with relaxed precision (Method 2). BP was recorded at Cz, C3, and C4 electrodes, with electroencephalogram (EEG) data averaged from -3 seconds to +1 seconds relative to electromyogram (EMG) onset. Method 1 required controlled wrist extensions with visual feedback, while Method 2 permitted varied movement parameters. BP parameters analyzed included peak amplitude, early slope, late slope, and onset time, compared across the two methods using paired t-tests.

Results: BP amplitude and slope were significantly greater during Method 1 than Method 2, particularly at the Cz and C3 sites. At Cz, all BP parameters were significantly higher in Method 1 compared to Method 2, including early slope (p = 0.0005), late slope (p = 0.0124), amplitude (p = 0.00003), and onset time (p = 0.0020). At C3, early slope (p = 0.0137), late slope (p = 0.0282), and amplitude (p = 0.0005) were significantly higher, while onset time showed no difference (p = 0.5823). At C4, only amplitude showed a significant difference (p = 0.034), with other parameters not reaching statistical significance. These findings indicate that Method 1, requiring precision, engaged motor cortical regions more intensively, particularly in preparatory neural activity.

Conclusion: This study highlights BP sensitivity to movement precision, with distinct BP features linked to precise motor tasks. BP monitoring could be valuable in neurorehabilitation, especially for conditions affecting motor preparation, such as Parkinson’s disease and post-stroke patients. Future research may expand BP applications, emphasizing its potential as a biomarker for motor preparedness and precise motor control interventions.

## Introduction

Motor control of voluntary movement involves a well-orchestrated activity within the higher order and execution mediating motor cortical areas, basal ganglia, and cerebellum [1,2]. Many studies have shown that these motor areas are activated about one to two seconds prior to the actual onset of movement [3-6] and are involved in the readiness or preparatory phase of movement. One of the simplest and non-invasive methods to study this readiness is by averaging EEG activity prior to each voluntary movement [5,7]. This activity prior to movement onset is called Bereitschaftspotentials (BP) [5]. Recording the cortical activity in the form of BP can be used as a tool to understand the supraspinal influences on motor planning [8].

Indeed, BP components and amplitude are decreased in neurodegenerative diseases, such as Parkinson's disease, where dysfunctional basal ganglia circuitry affects activity in the motor cortical areas [6,7]. Although the cortical substrates of planning are fairly known, the exact temporal or computational model of motor planning is far from understood. This is because motor planning involves a multitude of parameters like the selection of appropriate muscles or muscle groups, the selection of direction, force, range, and duration of movement. Together, these factors result in precise and desired movement. Such precision is achieved by regular feedback from subcortical areas like basal ganglia and cerebellum [2]. Studies show that the amplitude of BP positively correlates with the force, rate of contraction, and EMG response of the muscle involved in the motor task [9-11]. However, how the precision of movement (as a whole) is represented in components of BP is still not known.

In this study, using a unique recording paradigm, we aim to study the effect of precision on components of BP. Understanding these components can provide insights into the neural mechanisms underlying movement preparation and motor control, which may have implications for conditions affecting voluntary movement, such as Parkinson's disease.

## Materials and methods

Study design and participants

The study employed a quasi-experimental design to investigate the effects of precision in movement on BP. The study was conducted at All India Institute of Medical Sciences, New Delhi, in the Cognitive Neurophysiology Lab of the Department of Physiology from January 2012 to December 2013. The study received approval from the Institutional Ethics Committee (IESC/T-93, 2011) and was conducted in compliance with the ethical principles outlined in the World Medical Association's Declaration of Helsinki (2000). Participants were well informed about the purpose of this study, and written informed consent was taken from each following standard institutional ethical guidelines. Confidentiality was maintained by encrypting and securely storing the participant data. Healthy male participants aged 40 to 60 years were included in this study. Inclusion criteria required participants with no history of neurological, psychiatric, or musculoskeletal disorders, normal or corrected-to-normal vision and hearing, and not taking any medications affecting the nervous system. Only right-handed participants as screened using the Oldfield questionnaire [12] were selected. We excluded individuals with any history of head trauma, stroke or any other neurological complications, or psychiatric disorders. A post-hoc power analysis using a paired t-test was conducted for BP components at Cz with an alpha level of 0.05 and a sample size of 15. The results showed high power for the peak amplitude (0.99999), early slope (0.996), and onset time (0.983), while late slope (0.898) had slightly lower but still acceptable power. These findings indicate that the study was well-powered to detect differences in BP components, particularly for peak amplitude, early slope, and onset time. However, future studies with a larger sample size could further enhance power, especially for late slopes.

Recording setup

The participants were seated comfortably on armchairs throughout the recording. They were instructed to pay attention to the screen kept 60 cm away and minimize eye blinking during the task. The potentials were recorded using Neuropack 8 (Evoked Potential Recorder Nihon Kohden, Japan) at electrode sites Cz, C3, and C4 using the standard international 10-20 system. Electroencephalography was recorded from the scalp at the above-mentioned sites using Ag/AgCl surface electrodes with linked earlobe electrodes as a reference and a forehead electrode (Fpz) used as the ground. The potential recorder has in-built amplifiers that result in a signal gain of 10,000× (or 10,000-fold). The EEG signal was filtered using a bandpass filter ranging from 0.05 to 45 Hz to avoid high-frequency disturbances in the data. The EEG signal (3.0 seconds prior to and 1.0 seconds after the EMG onset) was back averaged using the onset of EMG as the trigger for averaging. Electromyography (EMG) was recorded from the extensor carpi radialis muscle with two recording electrodes placed 2 cm apart. EMG signal was filtered using 0.05-3kHz bandpass. A notch filter of 50 Hz was used for all recordings. In addition, eye blinks were recorded from Fp1-A1 and Fp2-A2 electrode pairs. Electrode impedance was ensured to be less than 5 KΩ throughout the recording.

Experimental conditions

To study the effect of movement precision on BP, the participants were asked to perform the same task using two methods. In Method 1 (M1), the participants were trained to perform precise right wrist extensions (60 degrees from the horizontal position) once every five to 10 seconds, for a duration of 0.5 seconds. Before beginning the averaging process, each participant performed the wrist extension according to the given instructions, and their EMG waveform (amplitude and duration) was recorded. Visual feedback in the form of the EMG signal was provided on a screen, and the participants were instructed to ensure that the amplitude (maximum recorded during practice) and duration (0.5 seconds) of the signal remained consistent across all trials. In Method 2 (M2), the participants were instructed to perform self-paced right wrist extensions once every five to 10 seconds. No specific information was provided regarding the degree, duration, or amplitude of the movement, allowing the participants to vary these parameters. Although the precision of the movement was relaxed in M2, all movements in both methods were self-paced. Sweeps with eye blinks or EEG amplitudes exceeding 60 μV were excluded from the averaging process. For both methods, 100 artifact-free sweeps (trials) were averaged to obtain BP. Each participant performed both methods on separate days.

Data parameters and statistical analysis

Baseline activity was calculated by using EEG data from -3000 ms to -2500 ms, i.e., prior to the onset of BP in all recordings. The BP waveform morphology was analyzed for peak amplitude, early slope, late slope, and onset of BP according to our previous study [13]. Baseline activity was measured from -2500 ms to -2000 ms before the movement, with EMG onset set as time zero. The maximum amplitude, typically near -50 ms relative to the EMG onset, was noted as the peak amplitude. The early BP component was represented by the average slope from -1500 ms to -500 ms (early slope), while the late component was represented by the average slope from -500 ms to 0 ms (late slope) [14]. The intercept of the early slope with baseline represents the onset time [13]. The Kolmogorov-Smirnov test confirmed normality for all BP parameters, and they were compared between the two methods using a paired t-test in GraphPad Prism (version 8.0.1, GraphPad Software, Inc.) with an alpha level of 0.05.

## Results

**Figure 1:**
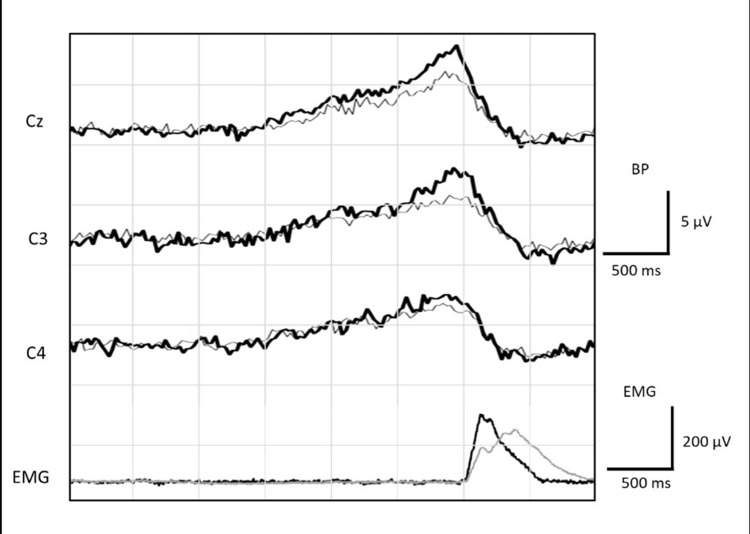
Grand average Bereitschaftspotential (BP) recordings for both methods This figure presents the grand average BP recordings for both methods at electrode sites Cz, C3, and C4. The rectified EMG signal is displayed below the BP traces. The vertical dashed line indicates the onset of the EMG signal, which was used as the trigger for back-averaging the EEG data from 3.0 seconds before to 1.0 seconds after the EMG onset. The black-colored graph represents Method 1 recordings, and the gray-colored represents Method 2 recordings.

**Figure 2:**
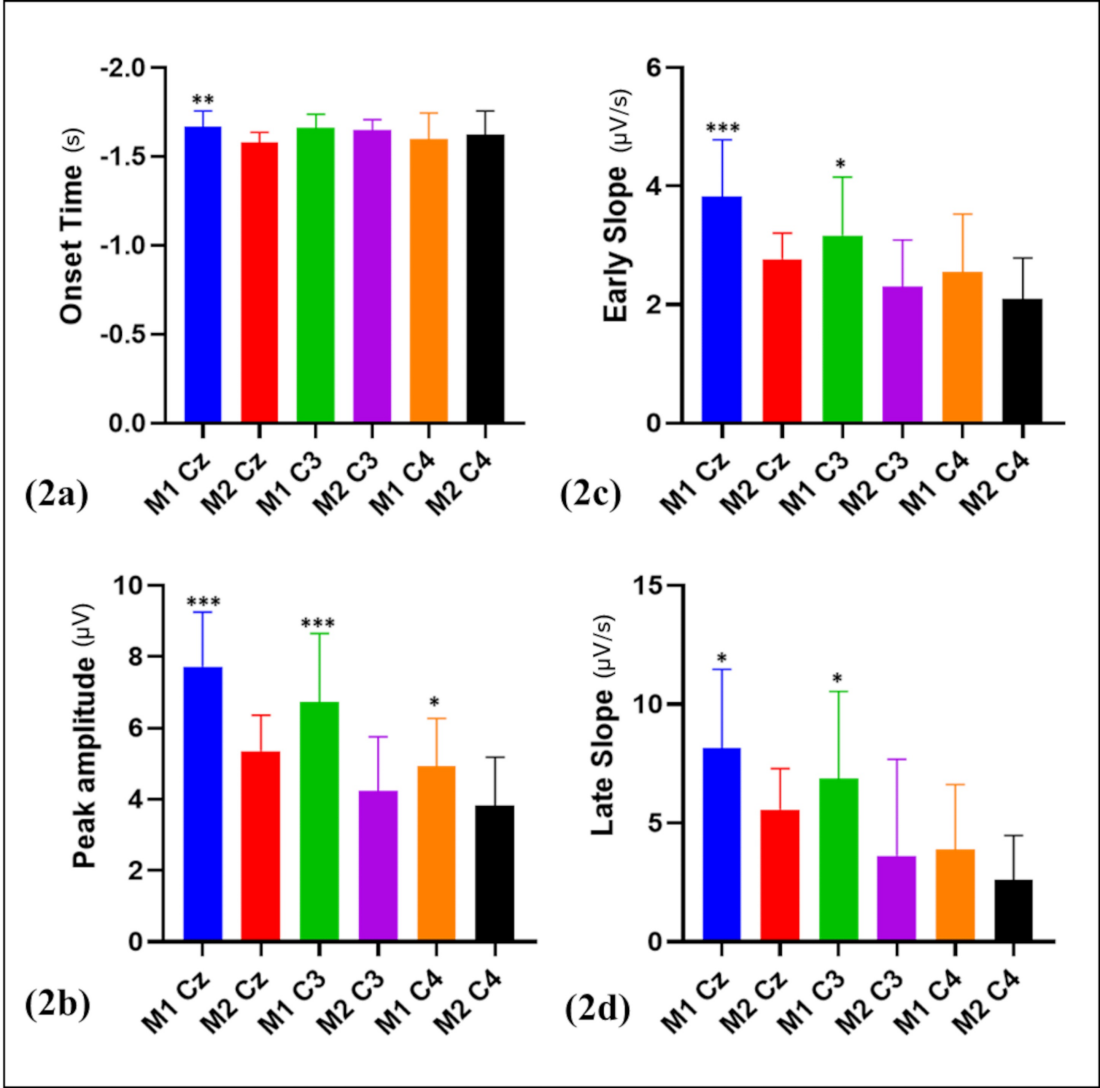
Comparison of Bereitschaftspotential (BP) parameters Comparison of BP parameters: (a) onset time, (b) peak amplitude, (c) early slope, and (d) late slope between the two methods, presented as mean ± SD. *p  < 0.05, **p < 0.01, ***p < 0.001.

We successfully recorded BP in 15 participants, with an average age of 51.47 ± 4.16 years, at electrode sites Cz, C3, and C4 with the two different methods. Figure 1 illustrates the grand average BP recordings for both methods at these electrode sites, accompanied by the rectified EMG. A visual comparison of these recordings indicates that BP amplitudes were greater when recorded during Method 1 compared to Method 2, suggesting that Method 1 elicited stronger neural responses in the preparatory phase of the movement. This observation is supported by the quantitative analysis presented in Figure 2, which compares four BP parameters between the two methods: (a) onset time, (b) peak amplitude, (c) early slope, and (d) late slope, with all values presented as mean ± standard deviation (SD) (Table 1).

**Table 1:** Bereitschaftspotential (BP) parameters between Method 1 and Method 2 across the electrode sites This table compares the BP parameters (early slope, late slope, peak amplitude, and onset time) recorded at electrode sites Cz, C3, and C4 during Method 1 vs. Method 2. Data are presented as mean ± standard deviation (SD), with corresponding p-values from paired t-tests.

Electrode site	Parameter (units)	Method 1 (mean ± SD)	Method 2 (mean ± SD)	p-value
Cz	Early slope (µV/s)	3.83 ± 0.95	2.77 ± 0.44	<0.001
Cz	Late slope (µV/s)	8.15 ± 3.32	5.57 ± 1.72	<0.05
Cz	Peak amplitude (µV)	7.73 ± 1.53	5.35 ± 1.01	<0.001
Cz	Onset time (s)	-1.67 ± 0.09	-1.58 ± 0.06	<0.01
C3	Early slope (µV/s)	3.17 ± 0.99	2.31 ± 0.78	<0.05
C3	Late slope (µV/s)	6.89 ± 3.66	3.62 ± 4.07	<0.05
C3	Peak amplitude (µV)	6.73 ± 1.93	4.24 ± 1.52	<0.001
C3	Onset time (s)	-1.66 ± 0.08	-1.65 ± 0.06	0.582268
C4	Early slope (µV/s)	2.55 ± 0.98	2.10 ± 0.69	0.151308
C4	Late slope (µV/s)	3.89 ± 2.73	2.59 ± 1.89	0.141524
C4	Peak amplitude (µV)	4.93 ± 1.35	3.83 ± 1.36	<0.05
C4	Onset time (s)	-1.60 ± 0.15	-1.62 ± 0.13	0.640892

Statistical analysis confirmed significant differences in BP parameters across all three electrode sites (Cz, C3, and C4) when comparing the two methods. At site Cz, all four BP parameters, i.e., early slope (p < 0.001), late slope (p <0.05), amplitude (p < 0.001), and onset time (p < 0.01), were significantly higher/earlier during Method 1 than Method 2. The early slope, which reflects the initial buildup of neural activity associated with motor preparation, and the late slope, representing activity closer to movement execution, both showed significant differences, indicating that Method 1 more effectively engaged motor preparation-related neural mechanisms. In addition, the significant increase in BP amplitude in Method 1 suggests a stronger overall motor preparation response. The earlier onset time further highlights that participants began preparing for movement earlier in Method 1, emphasizing heightened neural preparatory activity.

At site C3, significant differences were observed in early slope (p < 0.05), late slope (p < 0.05), and amplitude (p < 0.001), but not in onset time (p = 0.582268). These results suggest that Method 1 induces more pronounced neural activity in motor cortex regions at C3 (i.e., contralateral motor area for the movement), particularly in the buildup and magnitude of motor preparation. However, the lack of difference in the onset time at C3 indicates that while the magnitude of the neural response varies, the timing of motor preparation remains similar across both methods. At site C4 (i.e., ipsilateral motor area), the only significant difference observed was in BP amplitude (p < 0.05), with early slope (p = 0.151308), late slope (p = 0.141524), and onset time (p = 0.640892) showing no significant differences. This suggests that although Method 1 enhances neural responses in terms of amplitude, the timing and rate of neural activity buildup do not significantly differ between the methods.

To summarize, the analysis indicates that significant differences in BP parameters were primarily observed at sites Cz and C3, with all parameters showing significance at Cz and most at C3. At C4, only amplitude was significant, suggesting that method 1 elicits stronger motor preparation-related brain activity, particularly at Cz and C3.

## Discussion

The present study provides insights into how movement precision affects various components of the BP, revealing the role of cortical motor preparatory activity during voluntary movements. Our findings show that Method 1, which involved precise wrist extension, elicited significantly stronger BP characteristics compared to Method 2, in which movement precision was relaxed and random. Specifically, Method 1 was associated with a steeper early slope, larger BP amplitudes, and earlier onset time, particularly at the vertex (Cz) and contralateral motor area (C3) electrode sites. These findings suggest that tasks demanding precision may engage motor cortical regions more extensively, supporting previous research linking task complexity to increased BP activity and motor cortical involvement [15-20].

Previous studies indicate that BP amplitude is greater in complex tasks requiring simultaneous or sequential movements compared to simpler tasks [15], with activity localized primarily at the Cz electrode [16], reflecting the supplementary motor area’s (SMA) role in coordinating complex motor actions [17]. In addition, when tasks are more demanding, the SMA can be more active than the primary motor cortex [17], and BP onset appears earlier as task complexity increases. This pattern aligns with our finding of earlier BP onset and increased activation at the Cz electrode during Method 1, suggesting that motor preparatory processes are mobilized more intensively when precise movements are required. Voluntary movements are typically preceded by activation of the contralateral primary motor cortex, which follows initial SMA activity, with late component and peak BP originating primarily from contralateral motor regions [6,18-20]. This synergy of SMA and contralateral M1 activation underpins motor readiness, particularly when executing high-precision tasks [21]. The distinct BP characteristics observed in Method 1, including the pronounced early slope and higher peak amplitude, reflect enhanced neural engagement in both the SMA and contralateral motor cortex, emphasizing the role of motor synergies in facilitating precise movements. The early slope represents the rate of neural activity buildup preceding movement initiation, and our findings suggest that the precision demands of Method 1 activate motor cortical areas to a greater extent, a process supported by motor synergies that optimize neural coordination for challenging tasks. This reflects the SMA’s critical role in organizing coordinated activities across motor networks, which is crucial for high-precision tasks [6,22]. Further, the Cz electrode exhibited an earlier BP onset in Method 1, implying that motor preparation may start sooner when precision is required, aligning with studies indicating earlier SMA activation in complex tasks [15]. The lack of significant onset differences at C3 and C4 suggests that these regions may be less sensitive to movement precision, highlighting Cz’s unique role in engaging preparatory synergies for complex tasks [16].

Clinically, BP monitoring offers valuable insights into motor coordination and preparatory activity, with significant implications for neurodegenerative conditions like Parkinson’s disease (PD), where dopaminergic deficits disrupt motor planning and reduce BP amplitude and slope [13,23]. Neurofeedback training to enhance BP characteristics could improve motor coordination in PD by reinforcing motor circuitry [7], particularly in the SMA and contralateral M1. BP amplitude has been shown to increase with neurofeedback training, suggesting it could serve as a valuable indicator of motor preparedness [24]. Our results support BP monitoring as a tool for developing personalized rehabilitation strategies focused on precision and motor control. By measuring BP, patients can receive real-time feedback on their progress, facilitating improvements in motor planning and execution by engaging motor synergies. This feedback could benefit patients with motor impairments by reinforcing the focus on the motor preparation phase, which is essential for enhancing motor control over time [25]. Such motor precision paradigm may be tested on post-stroke patients during their recovery phase.

This study had certain limitations that should be acknowledged. First, the sample size was relatively small, with only 15 participants, which may limit the generalizability of the findings. Second, the study focused on an older population (40-60 years), and the results may not fully represent younger individuals. The quasi-experimental design, while informative, could be strengthened by a randomized controlled trial (RCT) to enhance the robustness of the conclusions. Furthermore, whole-brain mapping techniques could be employed in future research to explore additional brain regions involved in motor precision. Future studies may also investigate the dynamic interactions and functional connectivity patterns associated with varying levels of motor precision. Lastly, the potential application of BP in neurorehabilitation, particularly for conditions such as Parkinson’s disease and post-stroke recovery, warrants further exploration.

## Conclusions

Our findings emphasize BP’s sensitivity to movement precision, highlighting its potential as a biomarker for motor preparation and neurorehabilitation. Future studies examining the effect of movement precision on BP across neurological conditions may expand its clinical applications. This will enable the design of more personalized training protocols and provide new insights into the neural dynamics underlying motor preparedness in health and disease.
